# Deregulation of the miR-222-ABCG2 regulatory module in tongue squamous cell carcinoma contributes to chemoresistance and enhanced migratory/invasive potential

**DOI:** 10.18632/oncotarget.6253

**Published:** 2015-10-27

**Authors:** Luodan Zhao, Yuexin Ren, Haikuo Tang, Wei Wang, Qianting He, Jingjing Sun, Xiaofeng Zhou, Anxun Wang

**Affiliations:** ^1^ Department of Oral and Maxillofacial Surgery, First Affiliated Hospital, Sun Yat-sen University, Guangzhou, China; ^2^ Department of Gastroenterology, Nanfang Hospital, Southern Medical University, Guangzhou, China; ^3^ Department of Oral and Maxillofacial Surgery, Guanghua School of Stomatology, Sun Yat-sen University, Guangzhou, China; ^4^ Center for Molecular Biology of Oral Diseases, College of Dentistry, University of Illinois at Chicago, Chicago, IL, USA

**Keywords:** tongue squamous cell carcinoma, chemoresistance, cisplatin, metastasis, ABCG2

## Abstract

Chemoresistance is often associated with other clinical characteristics such as enhanced migratory/invasive potential. However, the correlation and underlying molecular mechanisms remain unclear. The aim of this study was to elucidate the function of the miR-222-ABCG2 pathway in the correlation between cisplatin (DDP) resistance and enhanced cell migration/invasion in tongue squamous cell carcinoma (TSCC). Using TSCC cell lines and primary cultures from TSCC cases, we first confirmed the correlation among DDP resistance (measured by IC50 values and ABCG2/ERCC1 expression), migratory/invasive potential (assessed by migration/invasion assays) and miR-222 expression. In TSCC cells, siRNA-mediated ABCG2 knockdown led to enhanced DDP responsiveness and reduced migratory/invasive potential, whereas ABCG2 overexpression induced DDP resistance and enhanced cell migration/invasion. Luciferase assays revealed that ABCG2 is a direct target of miR-222. In addition to reducing cell migration/invasion, functional analyses in TSCC cells indicated that miR-222 can reduce expression of the ABCG2 gene and enhance DDP responsiveness. However, co-transfection with ABCG2 cDNA restored both DDP resistance and migration/invasion. Moreover, miR-222 mimics and ABCG2 siRNA inhibited tumor growth and lung metastasis *in vivo*. Thus, our results verified that DDP resistance is correlated with enhanced migratory/invasive potential in TSCC. ABCG2 is a direct target of miR-222,and deregulation of the miR-222-ABCG2 regulatory module in TSCC contributes to both DDP resistance and enhanced migratory/invasive potential.

## INTRODUCTION

Tongue squamous cell carcinoma (TSCC) is the most common carcinoma in the oral and maxillofacial region and is characterized by rapid local invasion and migration. Cisplatin (DDP) is a standard chemotherapeutic agent that is effective for the treatment of TSCC, both as a single agent and in combination with other drugs [1]. Although treatment with DDP-based chemotherapy has been found to improve the prognosis of patients with TSCC, important clinical problems of DDP toxicity as well as intrinsic/acquired chemoresistance must be addressed [2, 3]. An even more serious issue was elucidated through a series of studies indicating that cancer cell migration and invasion are involved in the development of drug resistance [4, 5]. Indeed, many recent studies have found that chemoresistance and tumor migratory/invasive potential are the primary causes of treatment failure and mortality in cancer patients; however, the correlation and mechanisms remain unclear. Our previous study using TSCC cell lines revealed that DDP-induced chemoresistance caused the cells to undergo the epithelial-mesenchymal transition, a process that was accompanied by enhanced migration/invasion [6].

The ATP-binding cassette subfamily G member 2 (ABCG2) encodes a membrane transporter belonging to the ABC superfamily of membrane transporters, which are involved in the trafficking of biologic molecules across cell membranes [7]. Many studies have found that ABCG2 plays an important role in the efflux of xenobiotics, protecting cells against the damage caused by extraneous substances or drugs [8–10]. Furthermore, ABCG2 inhibitors enable the regulation of chemoresistance pumps and decrease cancer cell survival and are promising due to their minimal adverse effects to cancer patients [11]. Recent work has also suggested that ABCG2 might be closely associated with invasion and metastasis in tumors [7, 12]. For example, Xie et al. found that ABCG2 overexpression was responsible for chemotherapy failure, tumor recurrence and invasion in colon cancer [12]. MicroRNAs are essential regulators of diverse cellular processes, such as proliferation, differentiation, apoptosis, survival, motility, invasion and morphogenesis [13], and deregulation of microRNAs has been observed in many tumor types, including TSCC [14]. In our previous studies, numerous deregulated microRNAs, such as miR-7, miR-24, miR-99, miR-138, miR-181a and miR-222, were found to be related to migration/invasion in TSCC [13, 15–18]. miR-222 was found to reduce cell invasion by indirectly decreasing MMP1 expression by targeting SOD2 mRNA; thus, it might serve as a novel therapeutic target for TSCC patients at risk of metastatic disease [13]. Although several microRNAs that can target ABCG2, including miR-142-3p [19], miR-145 [20] and miR-487a [21], have recently been found, the relationship between miR-222 and ABCG2 has not yet been elucidated.

To investigate whether DDP resistance correlates with migratory/invasive potential in TSCC and whether this relationship is controlled by the miR-222-ABCG2 module, we first investigated the correlation among DDP resistance, as assessed by IC50 values and ABCG2/ERCC1 expression, migratory/invasive potential, as assessed by migration and invasion assays, and miR-222 expression in TSCC cell lines and primary cultural cells from TSCC cases. Next, we investigated the involvement of ABCG2 in DDP resistance and migration/invasion by ABCG2 knockdown and overexpression in TSCC cells. We determined that miR-222 directly targets ABCG2 in TSCC cells, resulting in enhanced DDP resistance and migratory/invasive potential. Finally, the effects of miR-222 and ABCG2 on tumor growth and lung metastasis *in vivo* were evaluated.

## RESULTS

### DDP resistance, migration/invasion and miR-222 expression in TSCC

**Figure 1:**
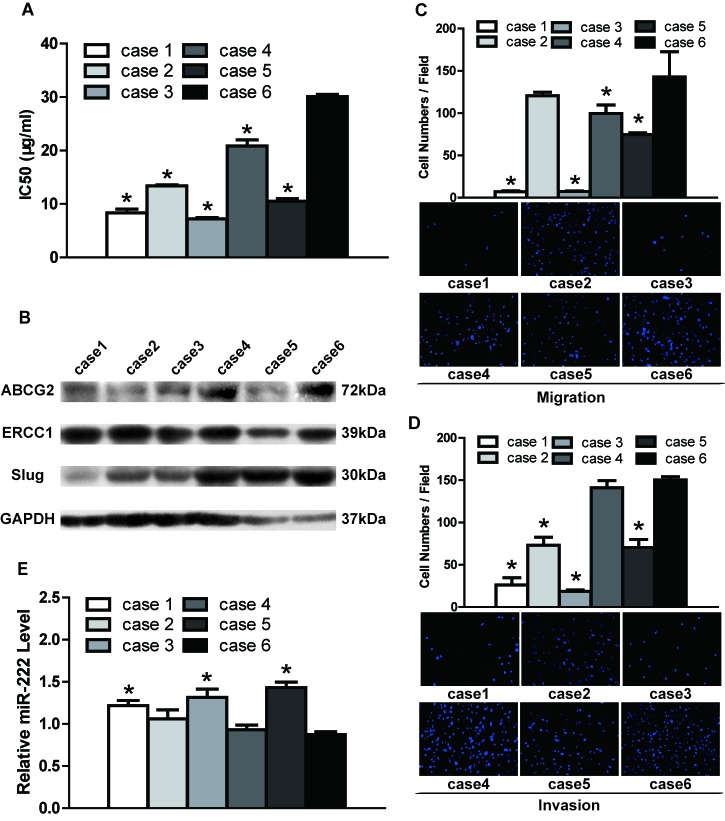
DDP resistance, migratory/invasive potential and miR-222 expression in TSCC primary cultural cells **A.** The IC50 values of DDP were detected by the CCK8 assay. Primary culture cells obtained from Case 6 had the highest IC50 values. **B.** Western blotting was used to detect the expression of chemoresistance markers (ABCG2 and ERCC1) and a metastasis-related gene (Slug). GAPDH was used as a loading control. The expression levels of ABCG2, ERCC1 and Slug were quantified; the results are shown in Figure S1. **C.** Relative cell migration was measured by the transwell migration assay. Primary cells obtained from Case 6 had significantly higher migration ability than those from Cases 1, 3, 4 and 5. **D.** Relative cell invasion was measured by the transwell invasion assay. Primary cells obtained from Case 6 exhibited a significantly higher invasion ability than Cases 1, 2, 3 and 5. **E.** The expression of miR-222 was measured by qRT-PCR. Primary cells obtained from Case 6 showed significantly lower expression of miR-222 than those from Cases 1, 3 and 5. *p < 0.05 vs. Case 6.

**Figure 2:**
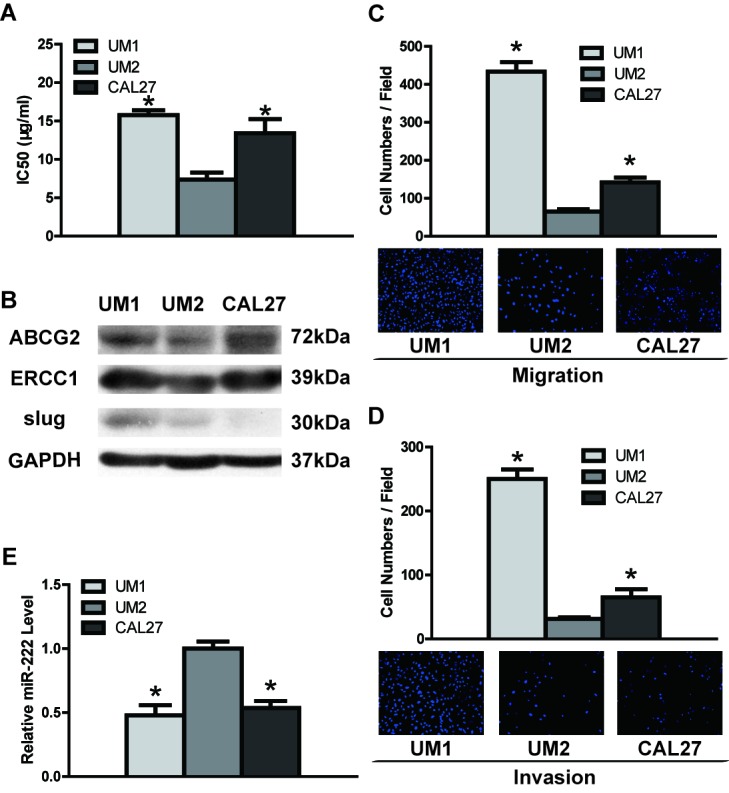
DDP resistance, migratory/invasive potential and miR-222 expression in TSCC cell lines **A.** UM2 cells had significantly lower IC50 values than UM1 and CAL27 cells. **B.** Western blotting was used to detected the expression of ABCG2, ERCC1 and Slug in TSCC cell lines. The results from western blots were quantified; the results are shown in Figure S1. (C and D) UM2 cells had significantly lower migration and invasion abilities than UM1 and CAL27 cells, as measured by transwell migration **C.** and invasion **D.** assays. **E.** UM2 cells showed significantly higher expression of miR-222 than UM1 and CAL27 cells, as measured by qRT-PCR. *p < 0.05 vs. UM2.

To investigate DDP resistance, DDP IC50 values and ABCG2/ERCC1 expression were assessed in TSCC cells. As shown in Figure 1A, the primary cultural cells obtained from Case 6 were the least sensitive to DDP, with the highest IC50 values compared to the primary cells obtained from the other five cases. The clinical characteristics of the TSCC cases are presented in Table S1. The lowest IC50 values were found for Cases 1 and 3, with no statistical significance between them, whereas mid-range IC50 values were found for Cases 2, 4 and 5 (Figure 1A, Table S2). As shown in Figure 1B, Figure S1A-S1B (quantification of western blot results) and Table S2, Case 6 exhibited the highest expression of ABCG2/ERCC1 (i.e., DDP resistance). Cases 1, 2, 3 and 5 displayed low ABCG2 expression and Case 4 mid-range expression; for ERCC1, Case 3 had the lowest expression and Cases 1, 2, 4 and 5 mid-range expression. With respect to TSCC cell lines, UM2 cells showed significantly lower IC50 values (Figure 2A, Table S3) and ABCG2/ERCC1 expression levels (Figure 2B, Figure S1C-S1D, Table S3) compared to UM1 and CAL27 cells. The exact *p* values between groups of TSCC primary cultural cells and cell lines are shown in Tables S2 and S3.

The migration and invasion potentials of TSCC cells are shown in Figure 1C–1D and Figure 2C–2D. Primary cells obtained from Case 6 (with higher DDP resistance) exhibited significantly higher migration activity compared to primary cells from Cases 1, 3, 4 and 5 (with lower DDP resistance) (Figure 1C and Table S2); Cases 1 and 3 exhibited the lowest migration activity, whereas mid-range migration activity was found for Cases 2, 4 and 5. In addition, Case 6 had significantly higher invasion potential compared to Cases 1, 2, 3 and 5, as shown in Figure 1D and Table S2; Cases 1 and 3 exhibited the lowest invasion potential and Cases 2, 4 and 5 mid-range potential. Regarding the TSCC cell lines, UM2 cells (with lower DDP resistance) had significantly lower migration/invasion activity than UM1 and CAL27 cells (with higher DDP resistance) (Figure 2C–2D, Table S3). Moreover, higher Slug expression (a metastasis-related gene) was found in Case 6 as well as UM1 cells, which are DDP resistant (Figure 1B–2B, Tables S2–S3).

Quantitative qRT-PCR was used to investigate the differential expression of miR-222 in TSCC cells. We found significant down-regulation of miR-222 in primary cells obtained from Case 6 compared to those from Cases 1, 3 and 5; the cells from these three cases exhibited the highest expression of miR-222. In contrast, cells from cases 2 and 4 showed mid-range miR-222 expression (Figure 1E, Table S2). With respect to TSCC cell lines, UM2 cells (with lower DDP resistance and migratory/invasive potential) had significantly higher miR-222 expression compared to UM1 and CAL27 cells (with higher DDP resistance and migratory/invasive potential) (Figure 2E, Table S3).

The above results revealed that cells with higher DDP resistance (higher IC50 values and ABCG2/ERCC1 expression) had higher migratory/invasive potential and lower miR-222 expression than cells with lower DDP resistance.

### Correlation among DDP resistance, migratory/invasive potential and miR-222 expression in TSCC

We further investigated a correlation among DDP resistance (IC50 values and ABCG2/ERCC1 expression), migration/invasion and miR-222 expression in TSCC cells. As shown in Table 1, strong correlations were found between IC50 values of DDP and ABCG2/ERCC1 expression levels, between IC50 values and migration/invasion potential, and between IC50 values and miR-222 expression. Moreover, strong correlations were found between migration/invasion potential and ABCG2/ERCC1 expression, between migration/invasion potential and miR-222 expression, and between ABCG2/ERCC1 expression and that of miR-222. Finally, strong correlations were found between migration and invasion potential and between the expression of ABCG2 and ERCC1 in TSCC cells. These results indicated correlations among DDP resistance, migratory/invasive potential and miR-222 expression in TSCC cells.

**Table 1:** Correlations among DDP resistance, migration/invasion potential and miR-222 expression in TSCC (Spearman)

	migration		invasion		ABCG2		ERCC1		miR-222	
	*r*	*p* value	*r*	*p* value	*r*	*p* value	*r*	*p* value	*r*	*P* value
IC50	0.767	0.000	0.887	0.000	0.723	0.000	0.599	0.001	−0.626	0.000
migration			0.823	0.000	0.509	0.007	0.580	0.002	−0.827	0.000
invasion					0.623	0.001	0.529	0.005	−0.630	0.000
ABCG2							0.747	0.000	−0.529	0.001
ERCC1									−0.524	0.005

### ABCG2 overexpression promotes DDP resistance and migration/invasion potential in TSCC

**Figure 3:**
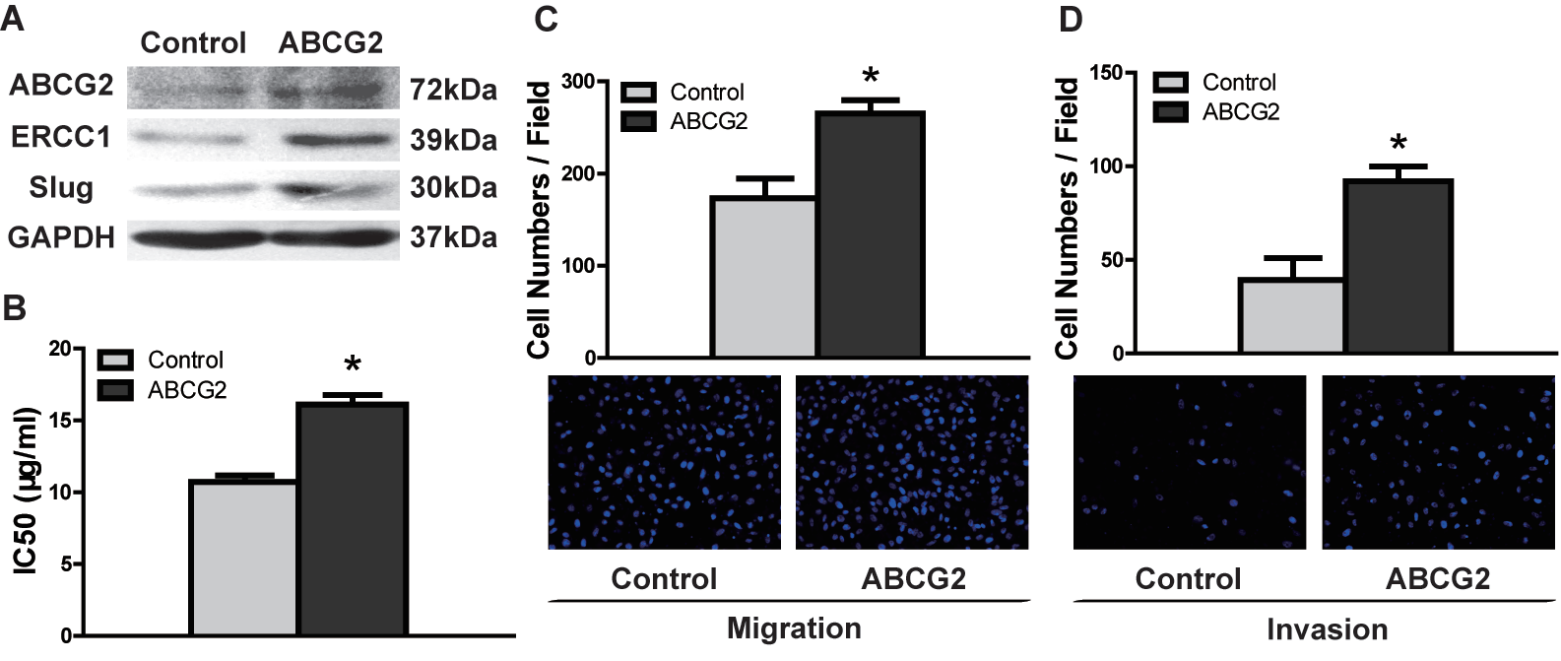
ABCG2 overexpression enhances DDP resistance and migratory/invasive potential in TSCC **A.** UM1 cells exhibited increased expression levels of ABCG2, ERCC1 and Slug proteins after transfection with ABCG2 cDNA, as detected by western blotting. **B.** UM1 cells had a significantly higher IC50 value after ABCG2 overexpression. (C and D) ABCG2 overexpression significantly increased the migration and invasion abilities of UM1 cells, as measured by transwell migration **C.** and invasion **D.** assays. *p < 0.05.

To investigate the effects of ABCG2 on DDP resistance and migration/invasion potential, we overexpressed ABCG2 in UM1 cells. The UM1 cell line was selected because it is more easily transfected with plasmid than UM2 cells (data not shown). ABCG2 protein abundance was significantly increased in UM1 cells after transfection with a plasmid containing ABCG2 cDNA (Figure 3A), and UM1 cells overexpressing ABCG2 displayed increased expression of ERCC1 and Slug (Figure 3A) and IC50 values of DDP (Figure 3B) compared to control plasmid-transfected cells. Furthermore, ABCG2 overexpression also resulted in increased migration/invasion abilities in UM1 cells (Figure 3C, Figure 3D). These results indicated that ABCG2 overexpression promoted DDP resistance and migration/invasion potential in TSCC cell lines.

### ABCG2 knockdown inhibits DDP resistance and migration/invasion in TSCC

**Figure 4:**
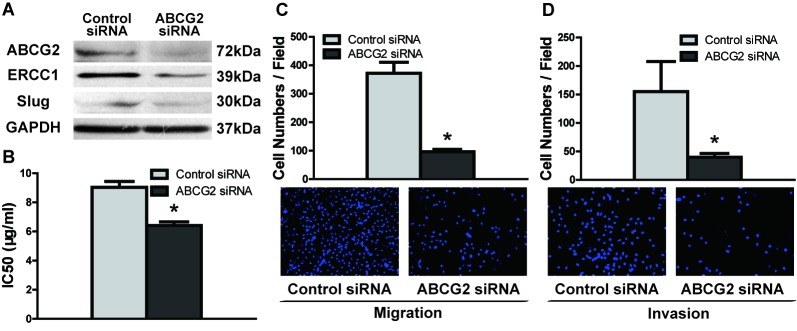
ABCG2 knockdown inhibits DDP resistance and migratory/invasive potential in TSCC **A.** UM1 cells exhibited decreased levels of ABCG2, ERCC1 and Slug proteins following ABCG2 knockdown, as detected by western blotting. **B.** UM1 cells had a significantly lower IC50 value after transfection with ABCG2 siRNA. (C and D) ABCG2 knockdown significantly inhibited the migration and invasion abilities of UM1 cells, as measured by transwell migration **C.** and invasion **D.** assays. *p < 0.05.

To investigate the function of ABCG2 in augmenting DDP resistance and migration/invasion potential, we knocked down the expression of ABCG2 using RNA interference. The protein level of ABCG2 was significantly decreased in UM1 cells after transfection with ABCG2 siRNA (Figure 4A), with decreased expression of ERCC1 and Slug (Figure 4A) and IC50 values of DDP (Figure 4B) compared to control siRNA-transfected cells. Furthermore, ABCG2 knockdown also resulted in decreased migration/invasion abilities (Figure 4C, Figure 4D) in UM1 cells. Similar results were also found in CAL27 cells after transfection with ABCG2 siRNA (Figure S2).

### The miR-222-ABCG2 pathway is related to DDP resistance and migration/invasion in TSCC

**Figure 5:**
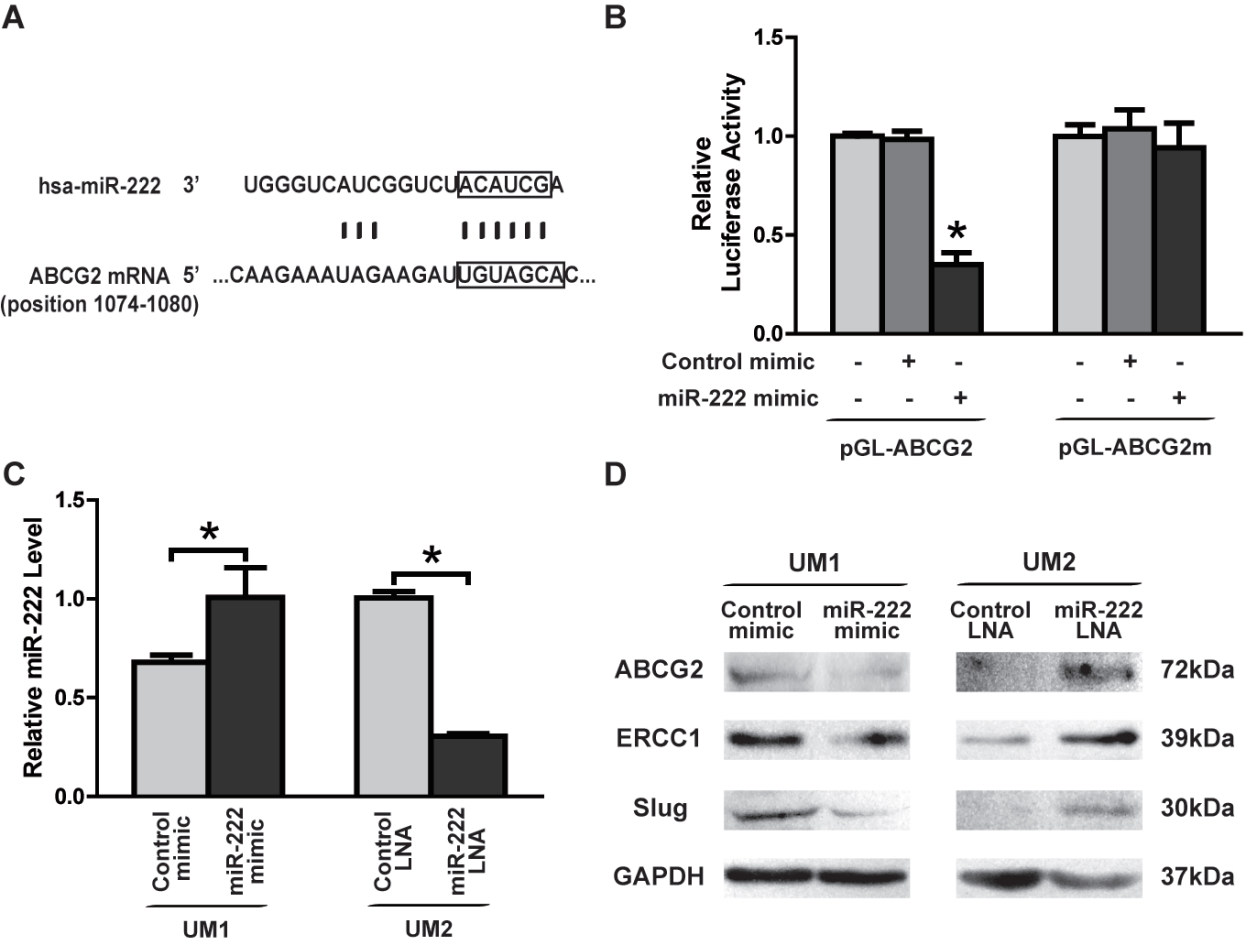
miR-222 directly targets and reduces the expression of ABCG2 in TSCC **A.** Predicted target sequences for miR-222 are located in the 3′-UTR of ABCG2 mRNA. **B.** Dual luciferase reporter assays were performed to evaluatethe target gene of miR-222. Following transfection with pGL-ABCG2 for 24 h, luciferase activity was significantly reduced in cells treated with miR-222 mimics relative to cells treated with control mimics (*p < 0.05 vs. blank and control). After the seed region of the target site was mutated (pGL-ABCG2 m), the effects of miR-222 on luciferase activity were abolished. **C.** Differential expression of miR-222 was tested by quantitative qRT-PCR in UM1 cells transfected with control mimics or miR-222 mimics and UM2 cells transfected with control LNA or miR-222 LNA (*p < 0.05). **D.** UM1 cells displayed decreased levels of ABCG2, ERCC1 and Slug proteins following treatment with miR-222 mimics, and UM2 cells display increased levels of ABCG2, ERCC1 and Slug proteins following treatment with miR-222 LNA.

A targeting site for hsa-miR-222 was identified in the 3′-UTR of ABCG2 mRNA (Figure 5A), and dual luciferase reporter assays were performed to confirm that miR-222 directly targets this sequence. As illustrated in Figure 5B, when cells were co-transfected with miR-222 mimics and luciferase reporter constructs carrying pGL-ABCG2, luciferase activity was significantly reduced relative to cells that were co-transfected with the constructs in conjunction with control mimics. Furthermore, the effect of miR-222 on luciferase activity was abolished when the seed regions of the targeting sites were mutated (pGL-ABCG2m).

**Figure 6:**
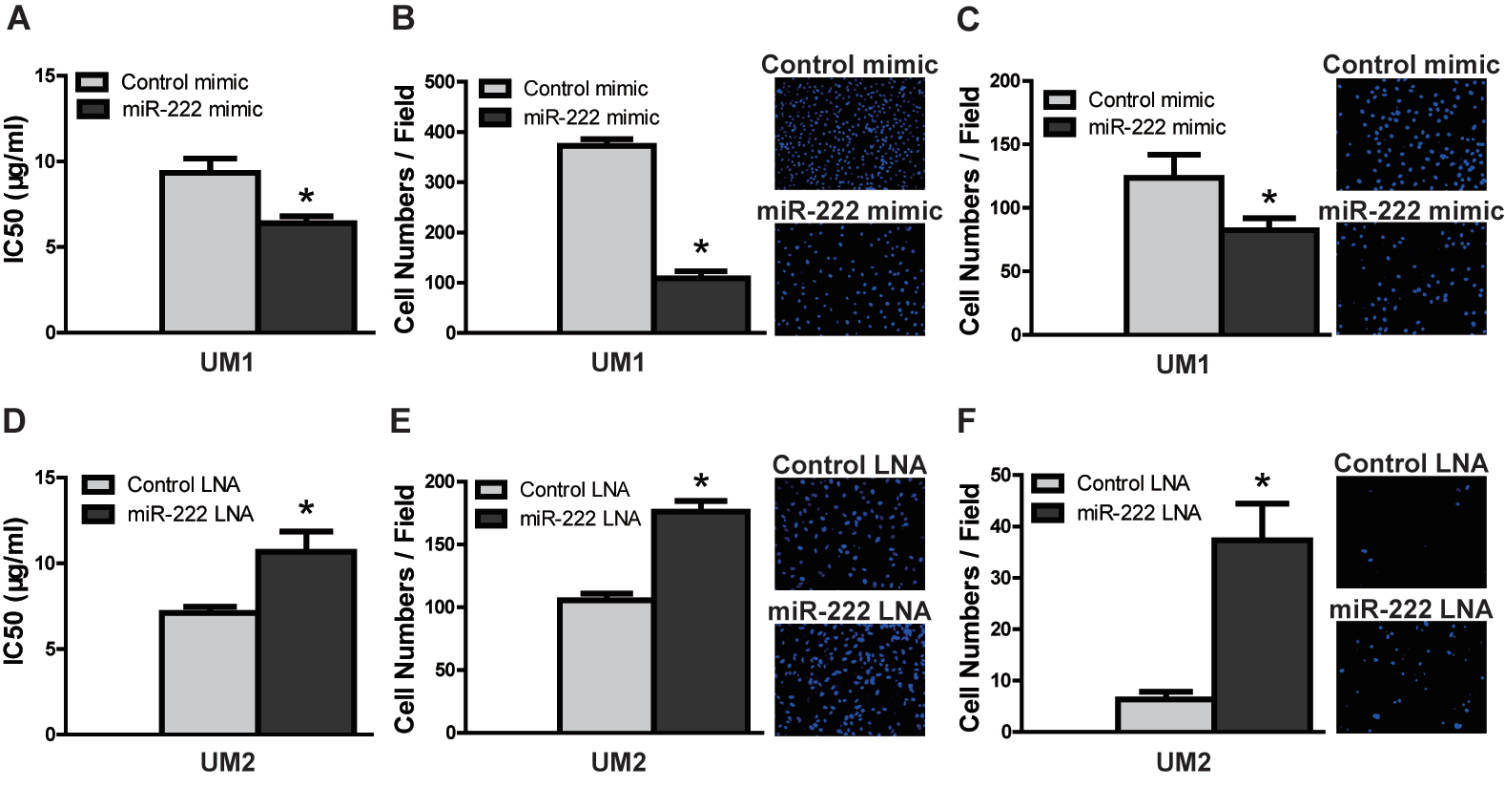
miR-222 reduces DDP resistance and migration/invasion in TSCC cells **A.–C.** UM1 cells were treated with either control mimics or miR-222 mimics for 24 h, and ectopic expression of miR-222 mimics led to significant decreasesin IC50 values **A.**, migration **B.**, and invasion **C.**. **D.–F.** UM2 cells were treated with either control LNA or miR-222 LNA for 24 h, and transfection with miR-222 LNA resulted in significant increases in IC50 values **D.**, migration **E.**, and invasion **F.**.*p < 0.05.

To confirm the role of the miR-222-ABCG2 pathway in TSCC, a functional analysis was performed to test the effects of miR-222 on DDP resistance and cell migration/invasion. Transfection of miR-222 mimics into UM1 cells led to a statistically significant increase in miR-222 levels (Figure 5C) that was accompanied by reductions in the expression of ABCG2, ERCC1 and Slug (Figure 5D). IC50 values of DDP (Figure 6A) and cell migration (Figure 6B) and invasion (Figure 6C) were all significantly decreased in UM1 cells following transfection with miR-222 mimics. Similar results were also found in CAL27 cells after transfection with miR-222 mimics (Figure S3). Moreover, when co-transfection with ABCG2 cDNA IC50 values (Figure S4A), expression of ABCG2, ERCC1 and Slug (Figure S4B), and migration/invasion potential (Figure S4C, Figure S4D) was increased in UM1 cells transfected with miR-222 mimics. Furthermore, when UM2 cells were treated with miR-222 LNA, a statistically significant decrease in miR-222 was observed. The expression levels of ABCG2, ERCC1 and Slug (Figure 5D), IC50 values (Figure 6D), migration (Figure 6E) and invasion (Figure 6F) were all significantly increased.

**Figure 7:**
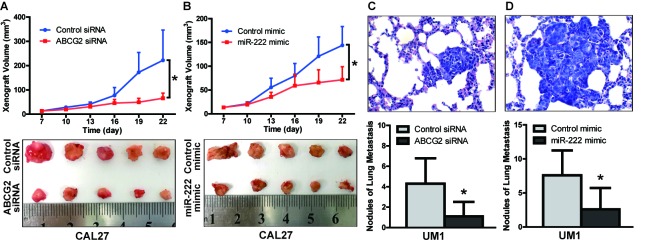
The miR-222-ABCG2 pathway regulates xenograft growth and lung metastasis of TSCC *in vivo* **A.** CAL27 cells stably infected with control siRNA or ABCG2 siRNA were inoculated subcutaneously into nude mice. Xenografts were measured every 3 days with a caliper, and growth curves demonstrated that ABCG2 knockdown significantly inhibited CAL27 xenograft growth. **B.** CAL27 cells stably infected with control mimics or miR-222 mimics were inoculated subcutaneously into nude mice. Growth curves demonstrated that miR-222 mimics significantly inhibited CAL27xenograft growth. **C.** UM1 cells stablyinfected with control siRNA or ABCG2 siRNA were injected into the tail vein of nude mice. Metastatic TSCC in the lung was assessed at week 6 after injection, as described in the methods. The histopathological analysis of lung metastasis (magnification 400×) and nodules of lung metastasis are shown. **D.** UM1 cells stably infected with control mimics or miR-222 mimics were injected into the tail vein of nude mice. The histopathological analysis of lung metastasis (magnification 400×) and nodules of lung metastasis are shown. *p < 0.05.

To further confirm the role of ABCG2 and miR-222 in the growth and metastasis of TSCC *in vivo*, xenograft growth and lung metastasis in nude mice were performed. CAL27 cells stably infected with lentivirus containing ABCG2 siRNA or miR222 mimics were inoculated subcutaneously into nude mice. As shown in Figure 7A, tumor growth was significantly suppressed in the group infected with ABCG2 siRNA relative to the group infected with control siRNA. The tumor doubling time was 3.6 days (control siRNA) and 6.6 days (ABCG2 siRNA), and the tumor inhibition rate on day 22 was 64.8% for the group treated with ABCG2 siRNA. As shown in Figure 7B, tumor growth was significantly suppressed in the group treated with miR-222 mimics relative to the group treated with control mimics. The tumor doubling time was 4.2 days (control mimics) and 5.8 days (miR-222 mimics), and the tumor inhibition rate on day 22 was 52.4% for the group treated with miR-222 mimics. UM1 cells stably infected with lentivirus containing ABCG2 siRNA or miR222 mimics were also injected into the tail vein of nude mice, and metastatic nodules in the lungs were confirmed histologically and counted. The mice injected with ABCG2 siRNA-infected cells exhibited a significantly reduced number of metastatic nodules relative to those injected with control siRNA-infected cells (Figure 7C), as did the mice injected with miR-222 mimic-infected cells relative to those injected with control mimic-infected cells.

Taken together, our data demonstrated that the miR-222-ABCG2 pathway is related to DDP resistance and migration/invasion potential in TSCC.

## DISCUSSION

Although DDP-based chemotherapy remains an effective method for the treatment of tongue cancer, chemoresistance has become a major chemotherapeutic obstacle. In the majority of studies, IC50 values following DDP treatment have been used to measure DDP resistance [22–24]: tissues with lower IC50 values are considered to be highly sensitive to drug treatment, whereas those with higher IC50 values are less sensitive. ABCG2 and ERCC1 can both serve as chemoresistance markers for most types of cancer [10, 25], and in the present study, we measured the IC50 values of DDP and the expression levels of ABCG2/ERCC1 to investigate DDP resistance in TSCC cells. We found that TSCC cells with higher IC50 values for DDP had significantly higher expression of ABCG2 and ERCC1 compared to TSCC cells with lower IC50 values. Furthermore, our data demonstrated a strong correlation of IC50 values with chemoresistance markers (ABCG2 and ERCC1). ABCG2 expression was also correlated with ERCC1 expression. The data confirmed that in addition to higher levels of ABCG2 and ERCC1 expression, TSCC cells with higher IC50 values are DDP resistant. Therefore, the combined detection of IC50 and ABCG2/ERCC1 expression may be highly effective at predicting DDP resistance.

Chemoresistance and tumor migratory/invasive potential are the main causes of treatment failure and mortality in cancer patients [26]. Recently, a series of studies indicated that migratory/invasive potential is involved in drug resistance in cancer cells: high migration/invasion activity was shown to enhance chemoresistance in tumor cells in pancreatic cancer, breast cancer, colorectal cancer and ovarian cancer [4, 27–29]. Many chemotherapeutic agents have been shown to induce migratory/invasive potential in several types of cancers [30–33], including head and neck carcinoma [31]. Although the above studies provide strong evidence of an association between chemoresistance and migratory/invasive potential, none have indicated a specific correlation between this potential and DDP resistance. In the present study, we found that TSCC cell lines as well as cells from cases exhibiting lower migratory/invasive potential were more highly sensitive to DDP (lower IC50 values and ABCG2/ERCC1 expression) than cells with higher migratory/invasive potential. Thus, a strong correlation between DDP resistance (IC50 values, ABCG2 or ERCC1 expression) and migration/invasion was also found. These findings suggest that cellular DDP resistance might be related to the migratory/invasive potential of TSCC, and novel strategies to fight against chemoresistance should be pursued to prevent enhanced migration/invasion.

ABCG2 has previously been found to play an important role in chemotherapy failure and migration/invasion in a variety of cancers, including colorectal [34], esophageal [11], and head and neck [35]. In human tissues, ABCG2 was found to be strongly expressed in OSCC but not in normal mucosa [36]. Additionally, it has been reported that ABCG2 expression is deregulated in tongue cancer and significantly associated with regional lymph node metastasis and local recurrence [10, 35, 36]. In the present study, we found ABCG2 expression to be strongly correlated with IC50 values, ERCC1 expression, and migration/invasion potential in both TSCC cell lines and primary cultures from TSCC cases. In TSCC cells, ABCG2 overexpression was found to increase IC50 values and expression of a chemoresistance-related protein (ERCC1); these cells concurrently exhibited increased migratory/invasive properties. In contrast, knockdown of ABCG2 in TSCC cells decreased IC50 values and ERCC1 expression, concurrent with reduced migration/invasion abilities. These data indicated that ABCG2 has an important function in DDP resistance and migratory/invasive potential in TSCC cells.

The mechanisms of miRNA-mediated target genes might contribute to various biological functions via multifactorial interactions that involve different biological pathways [37, 38]. In a previous study, we found that miR-222 plays an important role in TSCC invasion and might therefore serve as a novel therapeutic target for TSCC patients at risk of metastatic disease [13]. Although many target genes for miR-222 have been identified, including matrix metalloproteinase 1 (MMP1), superoxide dismutase 2 (SOD2), p27 [13], tissue inhibitor of metalloproteinase 3 (TIMP3) [39] and GNAI3 [40], few have been functionally implicated in both the chemoresistance and migratory/invasive potential of TSCC. However, our present study demonstrates that miR-222 directly targets ABCG2. A strong correlation between miR-222 expression and DDP resistance, as well as between miR-222 expression and migration/invasion was found. The up-regulation of miR-222 led to decreased IC50 values and induced the expression of ABCG2 and ERCC1, concurrent with decreased migration/invasion potential. In contrast, miR-222 knockdown in TSCC resulted in an increase in IC50 values, ABCG2/ERCC1 expression, and migration/invasion. Moreover, miR-222 mimics and ABCG2 siRNA significantly inhibited tumor growth and lung metastasis *in vivo*. These data reveal that miR-222-ABCG2 has an important function in the chemoresistance and metastatic potential of TSCC cells.

In consideration of the data presented above, we conclude that there is a correlation between IC50 values of DDP and ABCG2/ERCC1 gene expression. Therefore, concurrent detection of IC50 values and ABCG2/ERCC1 expression might constitute an effective method of predicting DDP resistance. In TSCC, DDP resistance is correlated with migration/invasion and miR-222 expression, which means that patients presenting with DDP resistance have more highly migratory/invasive cancer. Thus, alternative therapeutic regimens must be implemented for such patients. Furthermore, we demonstrated that ABCG2 is a direct target of miR-222 and that it plays an important role in chemoresistance and migration/invasion in TSCC. Thus, our data reveal the therapeutic target miR-222-ABCG2 regulatory module as a promising predictive signaling pathway for TSCC patients with high levels of DDP resistance and migration/invasion.

## MATERIALS AND METHODS

### Primary cultural cells

Archived tissue samples for primary cultural cells taken from six patients with TSCC were used in the present study. All of the patients underwent curative surgery, and none received any adjuvant therapy prior to surgery. The present study was approved by the Ethics Committee of the First Affiliated Hospital, Sun Yat-Sen University (2014-C-001). Among the TSCC cases, Case 6 had LN metastasis and experienced a recurrence of cancer after one month; this patient died seven months after surgery. In contrast, none of the other five cases were found to have LN metastasis, and Cases 1, 2, 4 and 5 had no recurrence during the 6-27 months of follow-up.

Primary cultural cells were maintained as previously described [41]. Briefly, TSCC tissues were disinfected with Betaisodona (Mundipharma, Limburg, Germany), rinsed twice in PBS, minced and placed in 2.5 mg/ml Dispase II (Roche, Mannheim, Germany) in DMEM for 18 to 24 h at 4°C. Subsequently, the tissues were incubated in 0.25% trypsin/EDTA for 5 min; trypsin activity was stopped using DMEM containing 10% fetal bovine serum (FBS). Tissue suspensions were centrifuged, resuspended in DMEM/F12 (containing 10% FBS, 1000 U/ml penicillin and 500 μg/ml streptomycin), and cultured at 37°C with 5% CO_2_. All of the ensuing experiments with primary samples were performed within a few days after surgery.

### Cell culture and transfection

The UM1 and UM2 cells used in this study are paired cell lines of different metastatic potentials generated from a single patient with TSCC [13]. CAL 27 cells are a TSCC cell line resistant to DDP [42]. These cells were maintained in DMEM supplemented with 10% FBS, 100 U/ml penicillin and 100μg/ml streptomycin at 37°C in a humidified incubator with 5% CO_2_. For functional analysis, a plasmid containing ABCG2 cDNA (NM_004827) and a control plasmid (pEGFP-N1) (GeneChem, Shanghai, China) were used. ABCG2-specific siRNA, control non-targeting siRNA (Genepharma, Shanghai, China), miR-222 mimics, control mimics (Genepharma, Shanghai, China), locked nucleic acid (LNA) inhibitor for miR-222 (anti-miR-222 LNA), and control LNA (Exiqon, Vedbaek, Denmark) were all transfected into the appropriate cells using Lipofectamine Transfection Reagent (Invitrogen, CA, USA) according to the manufacturer's instructions. The sequences of ABCG2 siRNA, miR-222 mimics and miR-222 LNA are shown in Table S4.

### Chemoresistance detected by IC50 using CCK8 assays

Cells were plated in triplicate in 96-well plates at a density of 5×10^3^ cells/well. After 24 h of incubation, the medium was replaced with fresh medium either with or without various concentrations of DDP (Hospira, Mulgrave, Australia). Cell viability was detected 24 h later using a modified Cell Counting Kit-8 (CCK8) assay (Fanbo, Beijing, China) according to the manufacturer's instructions. IC50 values were defined as the concentrations resulting in a 50% reduction in growth compared to control cell growth, with higher IC50 values indicating higher chemoresistance potential [24].

### *In vitro* cell migration and invasion assays

Transwell assays were performed to evaluate cell migration and invasion abilities using BD BioCoat Control Cell Culture Inserts or BD BioCoat BD MatrigelTM Invasion Chamber, respectively [15]. In brief, cells were seeded into the upper Boyden chambers of cell culture inserts. After 24 h of incubation, cells adhering to the lower membrane were stained with DAPI in the dark, imaged and counted using an inverted microscope equipped with a digital camera. Three random fields were captured at 200× magnification.

### Western blot analysis

Western blots were performed as previously described [43] using antibodies specific for ABCG2 (Santa Cruz, CA, USA), ERCC1 (Atlas Antibodies, Stockholm, Sweden), and Slug (Cell Signaling Technology, Beverly, MA); GAPDH (Sigma-Aldrich, MO, USA) was included as a control. The results obtained from western blotting were quantified using Quantity One software (Bio-Rad, CA, USA); the results are shown in Electronic Supplementary Figure S1.

### Quantitative qRT-PCR analysis

The expression levels of miR-222 in TSCC cells were determined using a miRNeasy mini kit according to the manufacturer's protocol (Qiagen, Hildon, German), as previously described [15]. Quantitative qRT-PCR reactions were performed using a 7500 Sequence Detection System (Applied Biosystems, CA, USA). The relative microRNA levels were computed using the 2^−delta delta Ct^ analysis method, and U6 was used as an internal reference.

### Dual luciferase reporter assay

Dual luciferase reporter assays were performed to test for interactions between miR-222 and its target sequence, as previously described [44]. In brief, a luciferase reporter gene construct for ABCG2 (pGL-ABCG2) was created by cloning the 3′-UTR of ABCG2 [NM_004827, containing the miRNA-222 binding site (5′-UGUAGCA-3′)] into the Xho I and Not I sites of a pGL-3-Control firefly luciferase reporter vector (Promega, Wisconsin, USA). The corresponding mutant construct (pGL-ABCG2m) was created by replacing the seed regions of the miR-222 binding sites with 5′-ACATCGT-3′. These constructs were then verified by sequencing. Cells were transfected with the reporter constructs using Lipofectamine 2000 (Invitrogen, CA, USA). The pRL-TK vector (Promega, Wisconsin, USA) was co-transfected to serve as an internal control for normalization of the transfection efficiency. Luciferase activity was then determined using a GloMax 20/20 luminometer (Promega, Wisconsin, USA), as previously described [15].

### Tumorigenesis and metastasis in nude mice

To investigate the involvement of ABCG2 and miR-222 in tumor growth and metastasis in TSCC, CAL27 and UM1 cells stably infected with lentivirus harboring ABCG2 siRNA / Control siRNA or miR-222 mimics / Control mimics (GeneChem, Shanghai, China) were collected and resuspended in PBS. Stably infected CAL27 cells (1×10^7^/0.2ml) were inoculated subcutaneously into the right flank of 4-week-old female BALB/c nude mice (purchased from Hunan SJA Laboratory Animal Co. Ltd., Hunan, China). Tumor volumes were calculated as 0.5 × length × width^2^, and the tumor growth curve (y = A e ^kday^), tumor doubling times (ln2/k) and tumor inhibition rate were obtained as previously described [15]. For metastasis assays, the stably infected UM1 cells (2 × 10^6^ /0.2 ml) were injected into the tail vein of BALB/c nude mice. The animals were sacrificed after six weeks, and metastatic tumors in the lung were assessed as previously described [15]. No mice showed a notable toxic effect or body weight loss during the experiment. The animal study was approved by the ethics committee of our institution (2014-C-001).

### Statistical analysis

All statistical analyses were carried out using Statistical Package for the Social Sciences (SPSS, Chicago, IL), Version 13.0. Correlations between DDP resistance and cell migration/invasion were tested using Spearman's rank correlation. The data were analyzed with Student's t test to determine significance between two groups or one-way analysis of variance (ANOVA) to calculate significance when there were more than two groups. Values of *p* < 0.05 were considered to be statistically significant.

## SUPPLEMENTARY MATERIAL FIGURES AND TABLES


