# Supplementary material for: Deregulation of the miR-222-ABCG2 regulatory module in tongue squamous cell carcinoma contributes to chemoresistance and enhanced migratory/invasive potential
Source: Oncotarget. 2015 Oct 27;6(42):44538–50. doi: 10.18632/oncotarget.6253 (PMC4792574; doi:10.18632/oncotarget.6253)
Supplement: Supplementary file 1 [file oncotarget-06-44538-s001.pdf]

Deregulation of the miR-222-ABCG2 regulatory module in tongue squamous cell carcinoma contributes to chemoresistance and enhanced migratory/invasive potential

Supplementary Material

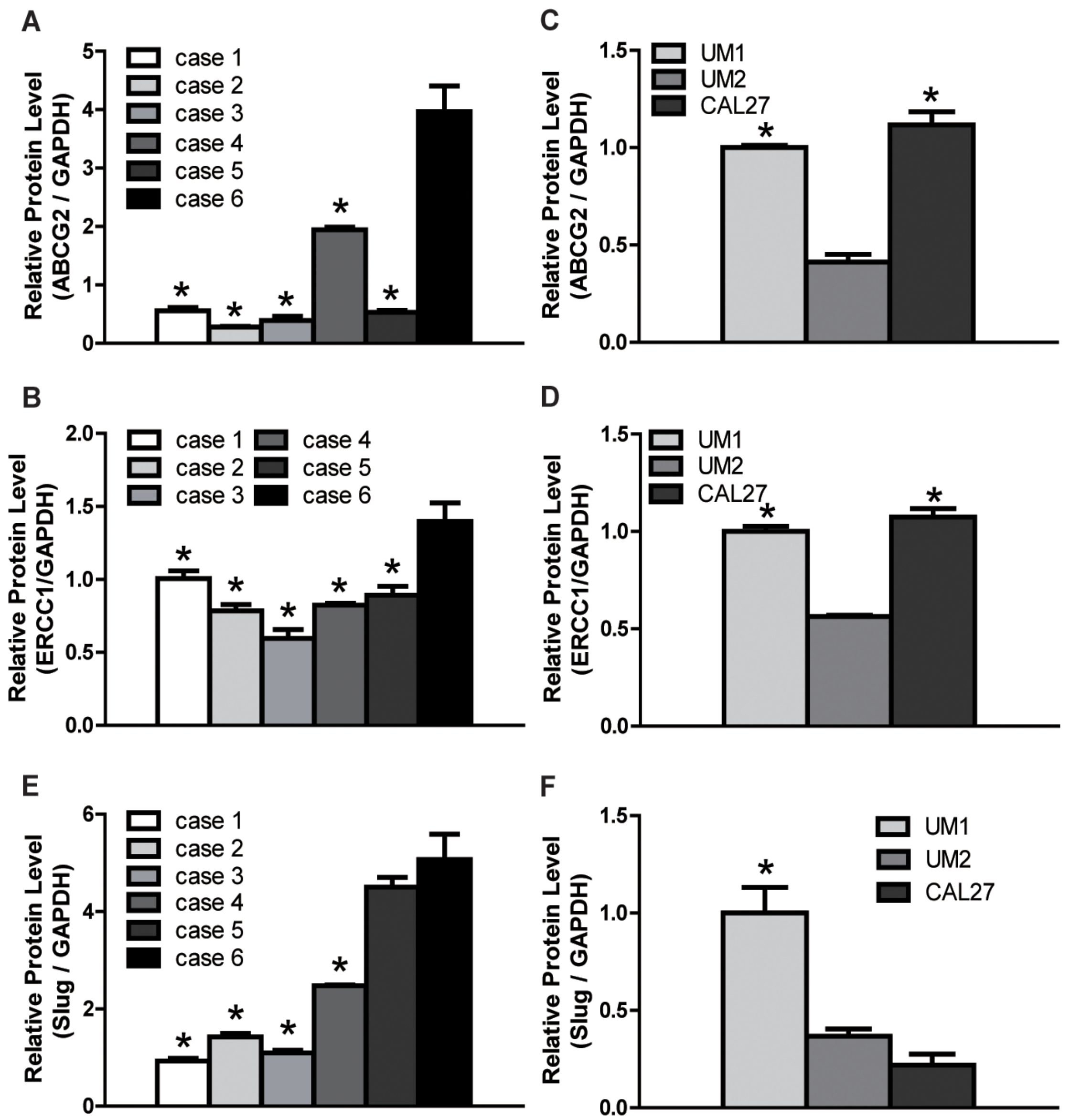

Figure S1: The expression levels of ABCG2, ERCC1 and Slug in Figures 1B and 2B were quantified using Quantity One software.

(A, B) (related to Fig. 1B) Case 6 demonstrated significantly higher levels of ABCG2 and ERCC1 expression compared to the other five cases (* $p<0.05$ vs. Case 6). **(C, D)** (related to Fig. 2B) UM2 had significantly lower levels of ABCG2 and ERCC1 expression than UM1 and CAL27 cells (* $p<0.05$ vs. UM2). **(E)** (related to Fig. 1B) Case 6 demonstrated

significantly higher levels of Slug expression compared to Cases 1, 2, 3 and 4 (* $p<0.05$ vs. Case 6). **(F)** (related to Fig. 2B) UM1 had significantly higher levels of Slug expression than UM2 and CAL27 cells (* $p<0.05$).

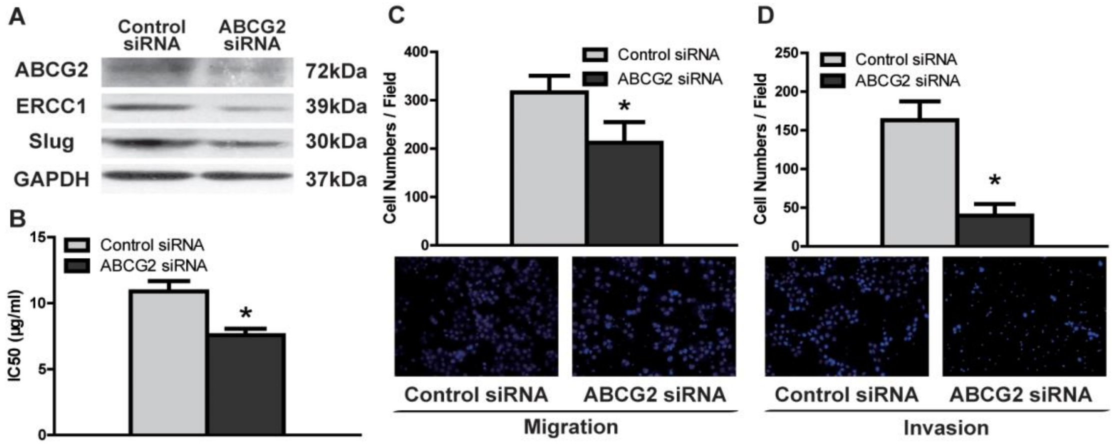

Figure S2: ABCG2 knockdown inhibits DDP resistance and migratory/invasive potential in CAL27 cells.

(A) CAL27 cells exhibited decreased levels of ABCG2, ERCC1 and Slug expression following ABCG2 knockdown, as detected by western blotting. **(B)** CAL27 cells had a significantly lower IC50 value following transfection with ABCG2 siRNA. **(C and D)** ABCG2 knockdown significantly inhibited the migration and invasion abilities of CAL27 cells, as measured by transwell migration (C) and invasion (D) assays. * $p<0.05$

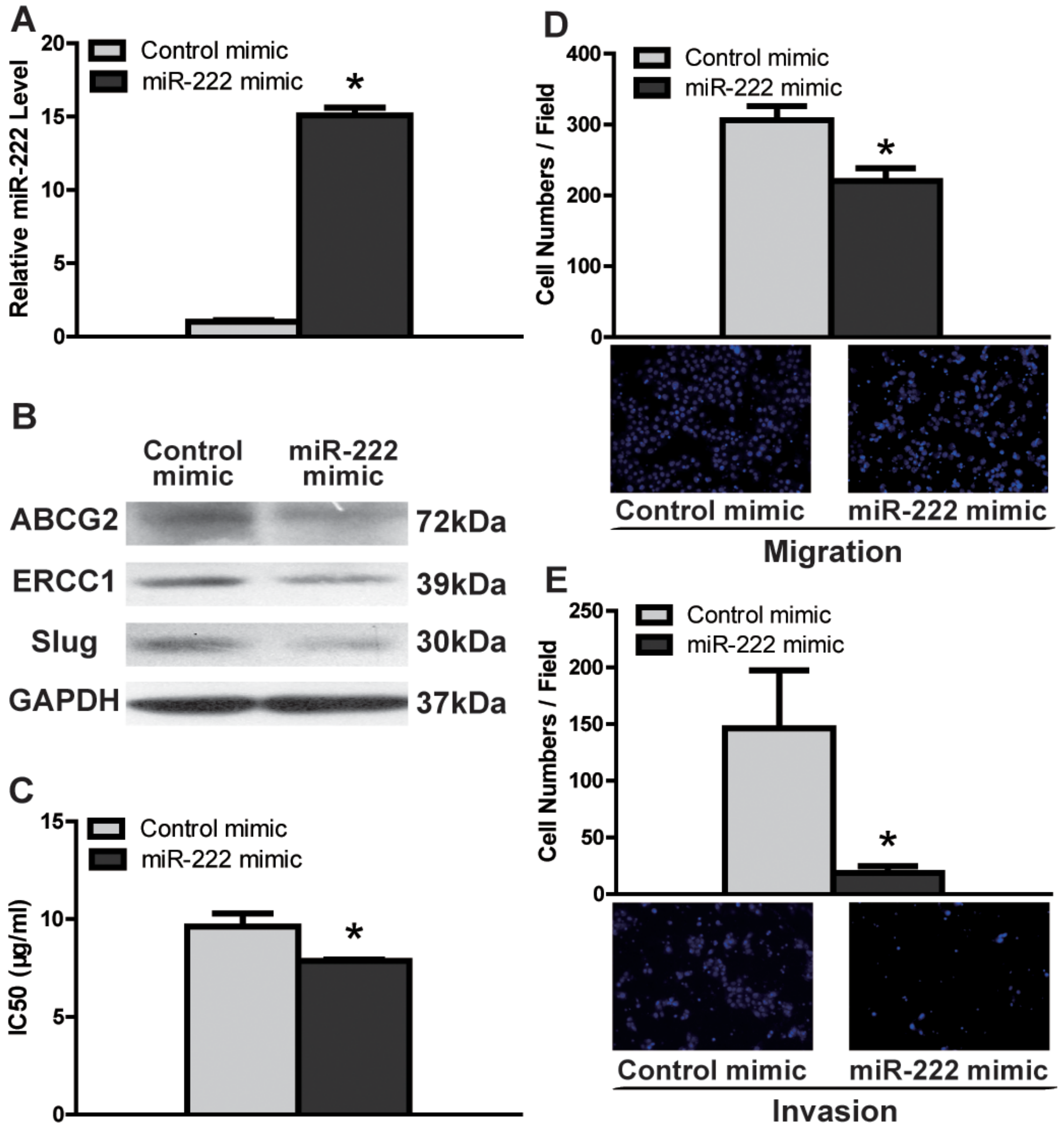

Figure S3: miR-222 mimics inhibit DDP resistance and migratory/invasive potential in CAL27 cells.

(A) CAL27 cells exhibited increased levels of miR-222 following transfection with miR-222 mimics, as detected by qRT-PCR. **(B)** CAL27 cells exhibited decreased levels of ABCG2, ERCC1 and Slug expression following transfection with miR-222 mimics, as detected by western blotting. **(C)** CAL27 cells had a significantly lower IC50 value following transfection with miR-222 mimics. **(D and E)** miR-222 mimics significantly inhibited the migration and invasion abilities of CAL27 cells, as measured by transwell migration (C) and invasion (D) assays. * $p<0.05$

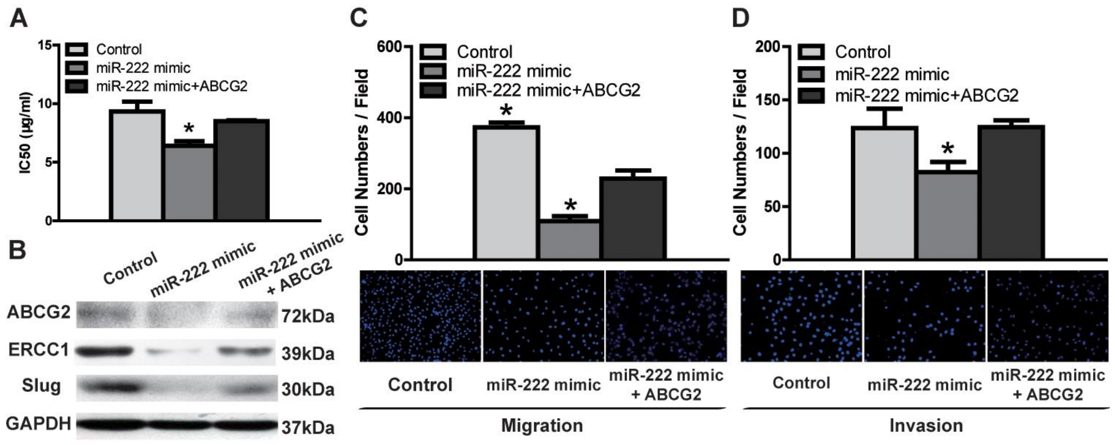

Figure S4: Co-transfection with ABCG2 cDNA restores DDP resistance and migration/invasion potential in UM1 cells transfected with miR-222 mimics.

(A) Co-transfection with ABCG2 cDNA increased IC50 values in UM1 cells transfected with miR-222 mimics. **(B)** Co-transfection with ABCG2 cDNA increased the expression of ABCG2, ERCC1 and Slug in UM1 cells transfected with miR-222 mimics. **(C and D)** Co-transfection with ABCG2 cDNA increased the migration and invasion abilities of UM1 cells transfected with miR-222 mimics, as measured by transwell migration (C) and invasion (D) assays. * $p<0.05$ vs. miR-222 mimics + ABCG2

Supplementary Tables

Table S1. Clinical characteristics of TSCC cases

Case	Gender	Age (ys)	TNM	Pathology diagnosis	LN metastasis	Recurrence	Follow-up
1	Female	46	T3N0M0	Low-D	N	N	27m
2	Male	48	T2N0M0	High-D	N	N	6m
3	Male	62	T2N0M0	Middle-D	N	4m ps	7m
4	Male	52	T1N0M0	High-D	N	N	6m
5	Male	57	T3N0M0	High-D	N	N	11m
6	Male	34	T3N2cM0	Middle-D	Y	1m ps	died 7m ps

Abbreviations: ys, years; ps, post-surgery; D, differentiation; Y, yes; N, no; m, months

Table S2. Exact *p* values compared between groups in TSCC primary culture cells (related to Fig. 1 and Fig. S1).

	Cases	Case1	Case2	Case3	Case4	Case5	Case6
IC50	Case1	0.000	0.000	0.576	0.000	0.015	0.000
(related to Fig. 1A)	Case2			0.000	0.000	0.001	0.000
	Case3				0.000	0.000	0.000
	Case4					0.000	0.000
	Case5						0.000
ABCG2	Case1	1.000	1.000	1.000	0.000	1.000	0.000
(related to Fig. 1B and Fig. S1A)	Case2			1.000	0.000	1.000	0.000
	Case3				0.000	1.000	0.000
	Case4					0.000	0.000
	Case5						0.000
ERCC1	Case1	0.029	0.029	0.000	0.106	0.985	0.000
(related to Fig. 1B and Fig. S1B)	Case2			0.085	1.000	1.000	0.000
	Case3				0.024	0.003	0.000
	Case4					1.000	0.000
	Case5						0.000
Migration	Case1	0.000	0.000	1.000	0.000	0.001	0.000
(related to Fig. 1C)	Case2			0.000	1.000	0.015	0.904
	Case3				0.000	0.001	0.000
	Case4					0.544	0.024
	Case5						0.001
Invasion	Case1	0.0	0.000	1.000	0.000	0.000	0.000
(related to Fig. 1D)	Case2			0.000	0.000	1.000	0.000
	Case3				0.000	0.000	0.000
	Case4					0.000	1.000
	Case5						0.000
Slug	Case1		0.312	1.000	0.000	0.000	0.000
(related to Fig. 1B and Fig. S1E)	Case2			1.000	0.002	0.000	0.000
	Case3				0.000	0.000	0.000
	Case4					0.000	0.000
	Case5						0.167
miR-222	Case1		0.363	1.000	0.007	0.057	0.001
(related to Fig. 1E)	Case2			0.018	0.780	0.001	0.133
	Case3				0.001	1.000	0.000
	Case4					0.000	1.000
	Case5						0.000

Table S3. Exact *p* values compared between groups in TSCC cell lines (related to Fig. 2 and Fig. S1).

		UM1	UM2	CAL27
IC50	UM1		0.001	0.182
(related to Fig. 2A)	UM2			0.003
ABCG2	UM1		0.000	0.060
(related to Fig. 2B and Fig. S1C)	UM2			0.000
ERCC1	UM1		0.000	0.067
(related to Fig. 2B and Fig. S1D)	UM2			0.000
Migration	UM1		0.000	0.000
(related to Fig. 2C)	UM2			0.004
Invasion	UM1		0.000	0.000
(related to Fig. 2D)	UM2			0.035
Slug	UM1		0.000	0.000
(related to Fig. 2B and Fig. S1F)	UM2			0.243
miR-222	UM1		0.000	0.944
(related to Fig. 2E)	UM2			0.000

Table S4. Sequences for miR-222 mimics, miR-222 LNA and ABCG2 siRNA

	Sequence
Control mimics	5’-UUCUCCGAACGUGUCACGUTT-3’
miR-222 mimics	5’-AGCUACAUCUGGCUACUGGGU-3’
Control LNA	5’-GTGTAACACGTCTATACGCCCA-3’
miR-222 LNA	5’-CCCAGTAGCCAGATGTAGC-3’
Control siRNA	5’-UUCUCCGAACGUGUCACGUTT-3’
ABCG2 siRNA	5’-GCCUACCUGAAAUUGUUAUTT-3’
